# Preferential enrichment and extraction of laser-synthesized nanoparticles in organic phases

**DOI:** 10.3762/bjnano.16.20

**Published:** 2025-02-20

**Authors:** Theo Fromme, Maximilian L Spiekermann, Florian Lehmann, Stephan Barcikowski, Thomas Seidensticker, Sven Reichenberger

**Affiliations:** 1 Technical Chemistry I and Center for Nanointegration Duisburg-Essen (CENIDE), University of Duisburg-Essen, Universitätsstrasse 7, 45141 Essen, Germany; 2 Department for Biochemical and Chemical Engineering, Laboratory for Industrial Chemistry, TU Dortmund University, Emil-Figge-Str. 66, 44227 Dortmund, Germany

**Keywords:** catalysis, laser ablation in liquid, laser synthesis and processing of colloids, phase transfer, size separation, thermomorphic multiphase system

## Abstract

Pulsed laser ablation in liquids (LAL) is an established preparation method of nanoparticles and catalysts, which additionally allows to chemically modify the nanomaterials in situ via chemical reactions of the nanoparticles with the molecules or solutes of the liquid. Particularly when organic solvents are used as liquids, photothermally induced C–C cleavage, addition or dehydrogenation reactions of the solvents, as well as (carbon) functionalization of the nanoparticles have been observed, which ultimately should affect their lipophilicity and, hence, colloidal stability in apolar or polar solvents. Two-phase liquid systems and the possibility to transfer the surfactant-free nanoparticles from one liquid phase into another remain practically unaddressed in literature. To tackle this knowledge gap, the present study investigates the phase preference of laser-generated noble metal (Au and Ag) and base metal (Cu, Fe, Al and Ti) nanoparticles within propylene carbonate/alcohol (PC/A) systems. Alcohols of increasing chain length (C_6_–C_11_) and hence decreasing polarity were chosen for this study. For each metal, LAL was performed at elevated temperatures (85 °C) where the PC/A mixture forms a single phase. Upon cooling, the phases separated and the amount of colloidal nanoparticles in the alcohol and propylene carbonate phase was analyzed for each metal system. The abundance of nanoparticles in PC or alcohol was found to correlate with the electrochemical reduction potential of the respective metal, where the noble metals were enriched within the more polar solvents. The polarity of the solvents (as function of the carbon chain length of the alcohol) was found to direct both the nanoparticles’ phase selectivity and recovery after cycling. The observed correlations provide potential guidelines for nanoparticle extraction and size separation, relevant for phase transfer and cycling during homogeneous catalysis.

## Introduction

Laser ablation in liquids (LAL) provides nanoparticles without the need of external surfactants while retaining the initial composition of the educt material in the formed nanoparticles [1–3]. However, the formed nanoparticles also interact with the used liquid during the process; thus, chemical reactions such as oxidation [3–6] or carbon shell formation [7–9] occur depending on the solvent’s properties, allowing for alterations of the structural properties and surface chemistry of the gained colloids. The use of organic solvents as liquid may result in reactive LAL processes [1,10] that cause elements from the solvent molecules (and solutes) to be part of the final nanoparticle’s composition. The solvent decomposition induced by laser-based nanoparticle synthesis was shown to produce gas phases consisting of hydrogen [11–13], carbon dioxide [12,14], and carbon monoxide [12,14], as well as carbon-based gases such as methane or C_2_ hydrocarbons [12–16]. In addition to gaseous by-products, the decomposition was found to produce liquid hydrocarbons such as pyrolysis products [15–18], polyynes [19–21], and dimers [13,22–23]. Furthermore, depending on the solvent and ablated material pairing, carbon may be “harvested” from the solvent forming crystalline carbides [24–27], amorphous carbon dopant [28–29], and/or carbon shells on the nanoparticle surface [7]. These carbon shells are either amorphous or graphitic [7–8,30], while doping of the shells [31] is also possible. Besides carbon formation, the choice of organic solvent influences the properties of the generated nanoparticles and process parameters. As such, nanoparticle size [32–33], colloidal stability [33], gas formation [11,34], degree of oxidation [35–37], and nanoparticle productivity [11,32–34] can be influenced and tailored to specific needs. Although it may be expected that the particles’ reactivity with the solvent and their surface chemistry will affect the particles’ wettability or hydrophobicity, the phase transfer between two liquid phases with different polarities has not been investigated previously. We approached this issue by using thermomorphic multiphase systems (TMSs), switchable mixtures that can change from a biphasic to a monophasic state depending on the applied temperature and have been widely studied in homogeneous catalysis [38–46]. The TMSs used in this study consist of a propylene carbonate (PC) phase (bottom) and an alcohol phase (top) and convert between the states at around 80 °C.

While laser-synthesized nanoparticles have been characterized regarding their (surface) oxidation [4–5,7] and carbon shells [7,24] previously, the phase preference of nanoparticles from laser synthesis and processing of colloids in liquid–liquid TMSs is unknown. However, to synthesize heterogeneous catalysts by deposition of nanoparticles, the specific nanoparticle solubility, and eventually also the colloidal stability of the individual nanoparticles, in different solvents is required [47–48]. If the colloid does not possess required properties such as colloidal stability, polarity of the solvent, or even physical attributes (e.g., boiling point), phase transfer methods are essential. To close this knowledge gap, we have performed ablation of six different metals (Au, Ag, Cu, Fe, Al, and Ti) ranging from high to low standard electrochemical reduction potentials in a propylene carbonate and 1-nonanol TMS and observed their phase preference to gain insights if the nanoparticle material has an impact on the preferred phase. We further narrowed down the influence of the TMS composition by varying the alkyl chain length of the alcohol from C_6_ to C_11_ and, consequently, changing the polarity of the non-polar solvent phase for the laser ablation of copper. Iron and copper stand in the middle of the investigated standard electrochemical reduction potential metal series and show quite interesting phase selectivity behavior. Moreover, cupreous nanoparticles are relevant for both heterogeneous and homogeneous catalysis [49–51]; hence, we investigated their phase selectivity in more detail. The accumulated Cu or Fe concentration in both phases was quantified and the TMSs were cycled through mono- and biphasic states repetitively to investigate the stability of the phase transfer process. Finally, the nanoparticles were analyzed to gain insights into the physical and chemical properties that determine the nanoparticle’s phase preference.

## Results and Discussion

### Influence of standard electrochemical reduction potential on phase preference

**Figure 1:**
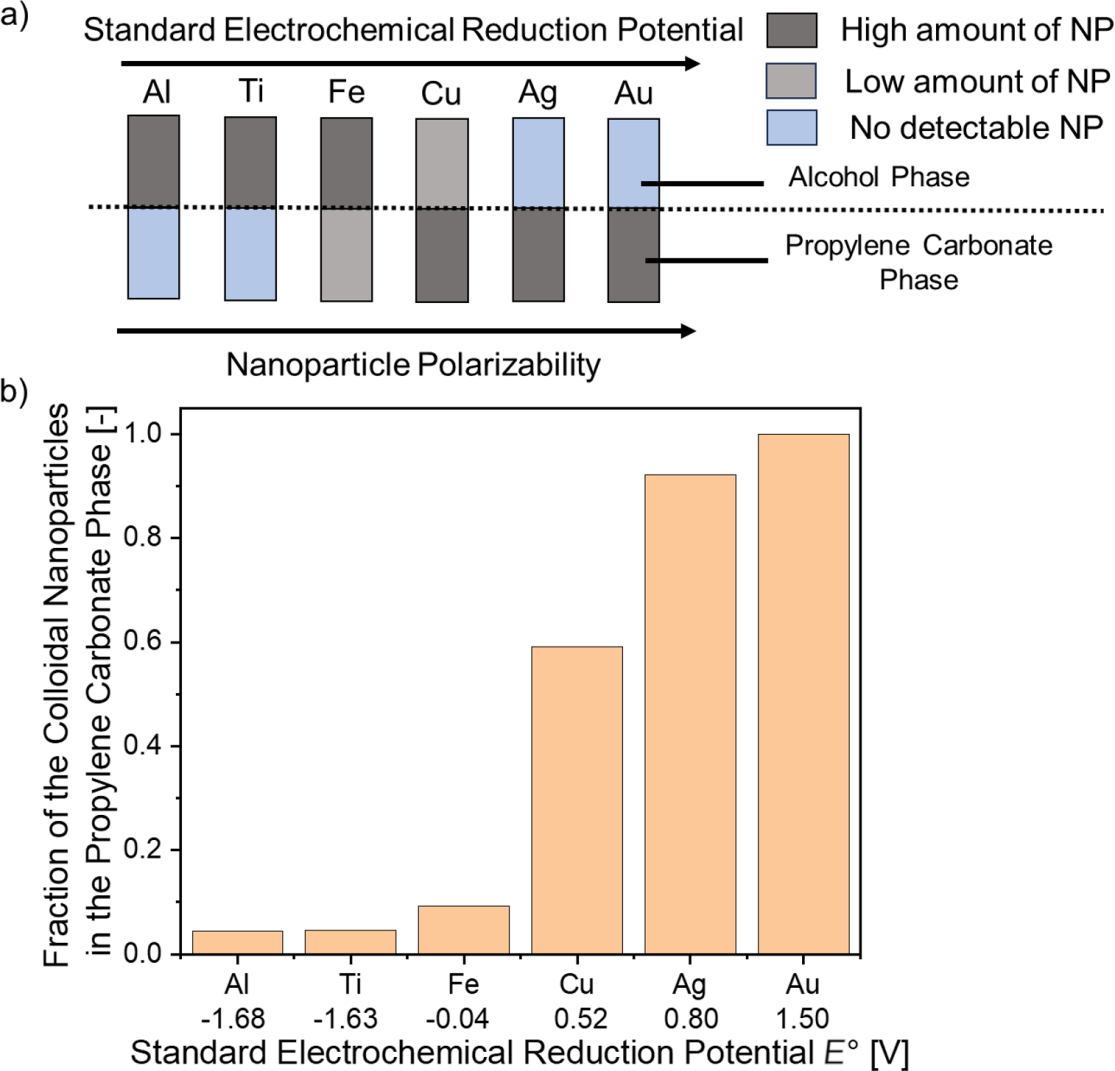
Study of the preferential accumulation of different metal nanoparticles synthesized by LAL at 85 °C in the single-phase TMS consisting of propylene carbonate and 1-nonanol after phase separation through cooling at room temperature. (a) Scheme of the six used metals ordered by the respective standard electrochemical reduction potential of the bulk material and the respective nanoparticle polarizability. The position of the nanoparticles is indicated by a gray coloring, while pure solvent is colored in blue. (b) Mass fraction of the gained colloidal nanoparticles in the propylene carbonate (bottom) phase of the TMS correlated with the standard electrochemical reduction potential *E*° for the respective metals.

Laser ablation of metals with varying standard electrochemical reduction potential (Au, Ag, Cu, Fe, Al, and Ti) was performed in the TMS of 1-nonanol and propylene carbonate under monophasic state conditions (85 °C). The gained colloids were cooled to room temperature by disabling the heating plate as it was observed that higher cooling rates (e.g., in an ice bath) would result in precipitation of the nanoparticle material in the interlayer of the two phases. After cooling to the biphasic state, the extinction of the gained colloidal phases was investigated by UV–vis measurements (Supporting Information File 1, Figure S1) to determine the preferred phase (1-nonanol or propylene carbonate) of the colloidal nanoparticles (Figure 1a,b). For gold and silver, the nanoparticles remained in the propylene carbonate phase, which is the more polar phase, while the nanoparticles made by LAL of aluminum and titanium were found to be in the less polar alcohol phase. In contrast to this behavior, copper and iron were not exclusively in one of the phases, but rather in both. With this, copper and iron represent the turning point in which the phase preference swaps from the more polar to the less polar liquid. Copper showed a higher mass concentration of nanoparticles in the more polar propylene carbonate phase, while iron preferred the less polar alcohol phase. If correlated to the standard electrochemical reduction potential *E*° (Figure 1b), a change in phase preference is visible after *E*° = 0 V is reached, resulting in noble metals accumulating in the propylene carbonate phase, while base metals gather in the alcohol phase.

The origin of this behavior is likely linked to the chemical composition of the nanoparticle surface. In earlier studies, nanoparticles of the more noble metals (gold, silver, and copper) formed by LAL in alcohols, ketones, or aliphatic hydrocarbons exhibited a carbon shell [7,11,52]. These carbon shells were particularly pronounced for gold and copper nanoparticles synthesized in hydrocarbons without a heteroatom. However, differences were found for the LAL in, for example, acetone. The LAL of gold in acetone did not lead to carbon shells, whereas the formation of carbon shells during the LAL of copper in acetone has been reported [35]. This observation was discussed to be linked to the catalytic activity of copper for C–C bond formation [53–54]. Accordingly, a stronger carbon formation is to be expected during the ablation of copper compared to gold and silver, which leads to a decreased polarity of the formed nanoparticles and potentially a higher affinity to less polar solvents. However, the base metals (iron, aluminum, and titanium) show a different behavior during LAL in organic solvents. Various nanoparticle compositions were found for iron [7,24–25] and titanium [26,55], including carbides, oxides, and oxocarbides, resulting in a higher affinity towards non-polar solvents. While this was not shown for aluminum nanoparticles previously, it is known that aluminum can bind enolates on its surface, which also yields a high affinity towards the non-polar alcohol phase [18].

**Figure 2:**
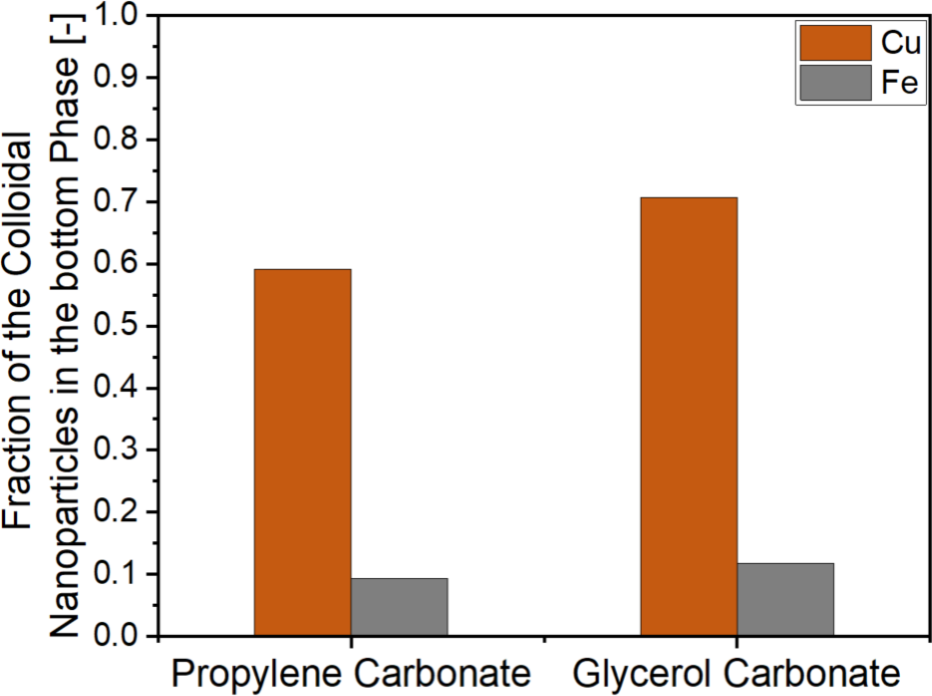
Comparison of the gained mass fraction in the bottom phase for the TMS consisting of either propylene carbonate or glycerol carbonate and 1-nonanol for copper and iron.

**Figure 3:**
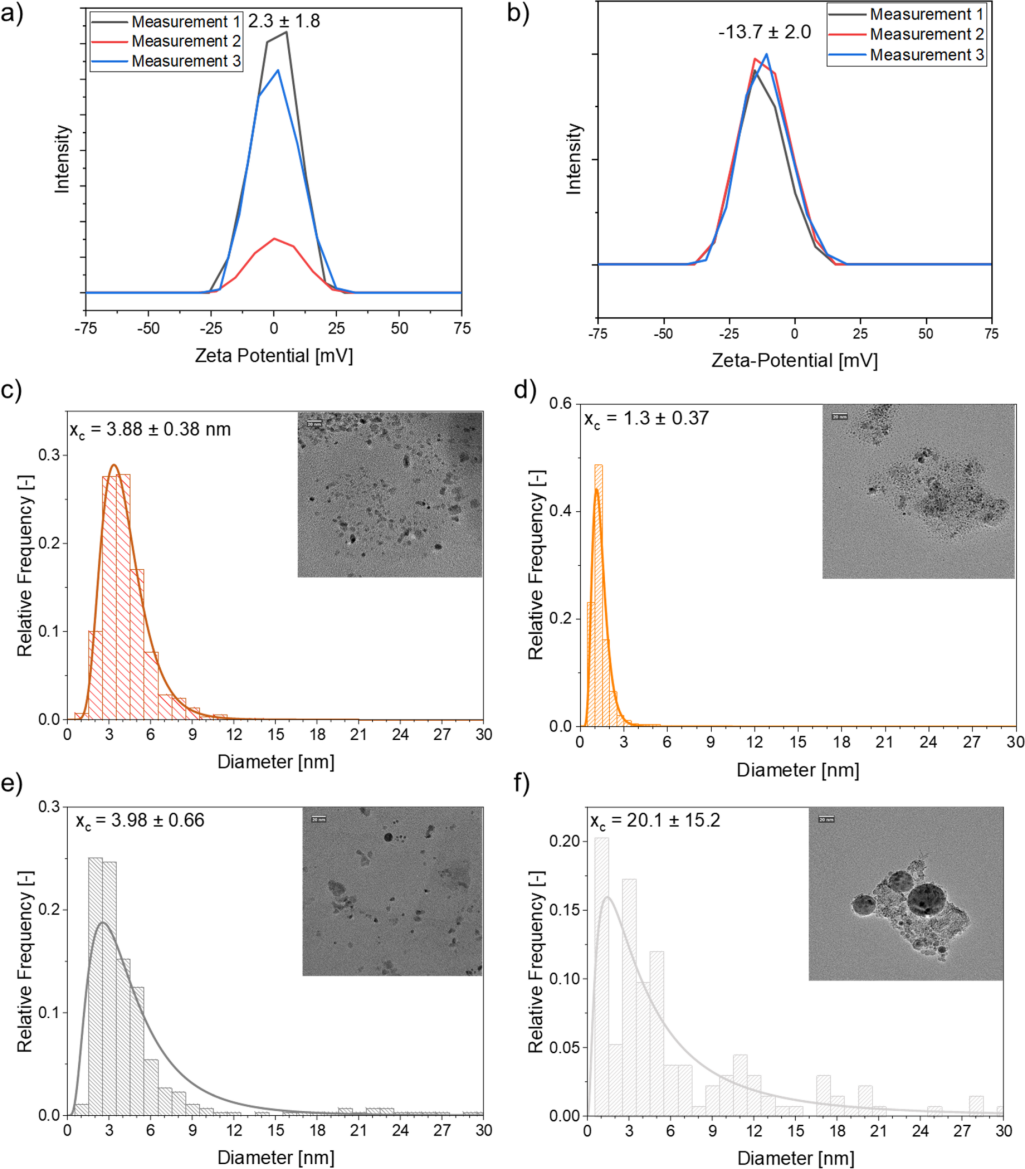
Zeta potential of copper nanoparticles in (a) 1-nonanol and (b) propylene carbonate obtained by LAL at 85 °C in the monophasic TMS of 1-nonanol and propylene carbonate. Size distribution and TEM images of the respective (c, d) copper and (e, f) iron nanoparticles in either (c, e) 1-nonanol or (d, f) propylene carbonate obtained by LAL in TMS.

The ligand effect of the alcohols on the different metal nanoparticles is another aspect affecting the colloidal stability that needs to be considered. The ligand effect is more pronounced for the base metals than for the noble metals. As nanoparticles produced by LAL of base metals tend to have a higher degree of surface oxidation, the alcohol groups can stabilize the nanoparticles electrostatically in the alcohol phase [56]. Yet, this effect should increase the affinity of the base metals to the more polar propylene carbonate phase. Investigation of this hypothesis was done by laser ablation of copper and iron in a TMS consisting of glycerol carbonate, which possesses another alcohol group and, hence, is also suited as a ligand, and 1-nonanol. After cooling, the fractions of the colloidal particles in the bottom phase (glycerol carbonate) increased for both metals (Figure 2). The fraction increases from 0.59 in propylene carbonate to 0.70 in glycerol carbonate and from 0.09 in propylene carbonate to 0.12 in glycerol carbonate for copper and iron, respectively. While the nanoparticle surface coverage with glycerol carbonate should be higher than for propylene carbonate, only a small increase for the fraction of iron particles (0.03) was visible, although they were assumed to be strongly oxidized. Instead, the mass concentration of copper in the more polar bottom phase increased, although the particles should be less oxidized. Hence, the properties of the bottom phase (to function as surface ligand) are influencing the mass fraction but do not change the preferred phase for nanoparticle enrichment. This also indicates that the alcohols functioning as ligands have no strong effect on the preferred phase of the nanoparticles either. The particle zeta potential is a third aspect that may affect the phase preference of the nanoparticles. Consequently, the zeta potential of the respective copper and iron colloids was measured for the colloids present in the PC and alcohol phases. While the particles in PC showed a negative zeta potential for copper and a fluctuating zeta potential ranging from negative to positive values for iron (Supporting Information File 1, Figure S2), the zeta potential of the particles in the alcohol phases was almost zero for both metals (Figure 3a,b). Further, a size dependency on the phase preference could be observed. The copper nanoparticles in propylene carbonate were smaller than those in the alcohol phase (Figure 3c,d), while the iron nanoparticles in the propylene carbonate phase were larger than those in the alcohol phase (Figure 3e,f). This could be attributed to the two simultaneously occurring and commonly accepted nanoparticle formation mechanisms happening during the plume phase of LAL (picosecond to longer nanosecond time scale) [57–58]. Recent spatiotemporal, large-scale molecular dynamic simulations show that the thermal history of nanoparticles depends on where in the plume they stem from, and there is a distribution of cooling rates ranging at least over three orders of magnitude, from less than 10^11^ to 10^13^ K·s^−1^, evidencing undercooling effects and defect-rich nanoparticle crystallization [59]. One may hypothesize that the different cooling rates also lead to different reactivity with the cooling solvent molecules that set the final surface chemistry of the particles and, thereby, affect their phase preference. Since the standard electrochemical reduction potential of Cu and Fe is close to 0 V, the different polarities of propylene carbonate and alcohol might influence the occurring interactions and reactions between the solvent and the Cu and Fe particles the most during nanoparticle formation, which then leads to a difference in phase preference of the formed nanoparticle fractions. Yet, this remains highly speculative and neglects that chemical reactions may also occur on longer timescales, during the cavitation bubble phase (microsecond time scale) [60]. The smaller iron nanoparticles are found in the alcohol phase, while the smaller copper nanoparticles prefer the propylene carbonate phase. Hence, another explanation for the phase preference could be a difference in post-irradiation behavior due to the use of a batch process. For example, it has been shown that the laser ablation of copper in ethylene glycol leads to nanoparticle diameters ranging from 2.5 to 4.8 nm [61]. Further research needs to investigate the correlations between the particle distribution, their size, and the particles’ degree of oxidation and crystal structure to differentiate the cause of this behavior, which is beyond this phase selectivity study. In this context, the systematic variation of the TMS via a systematic solvent chain length series is interesting.

### Tunability of phase preference by chain length variation

**Figure 4:**
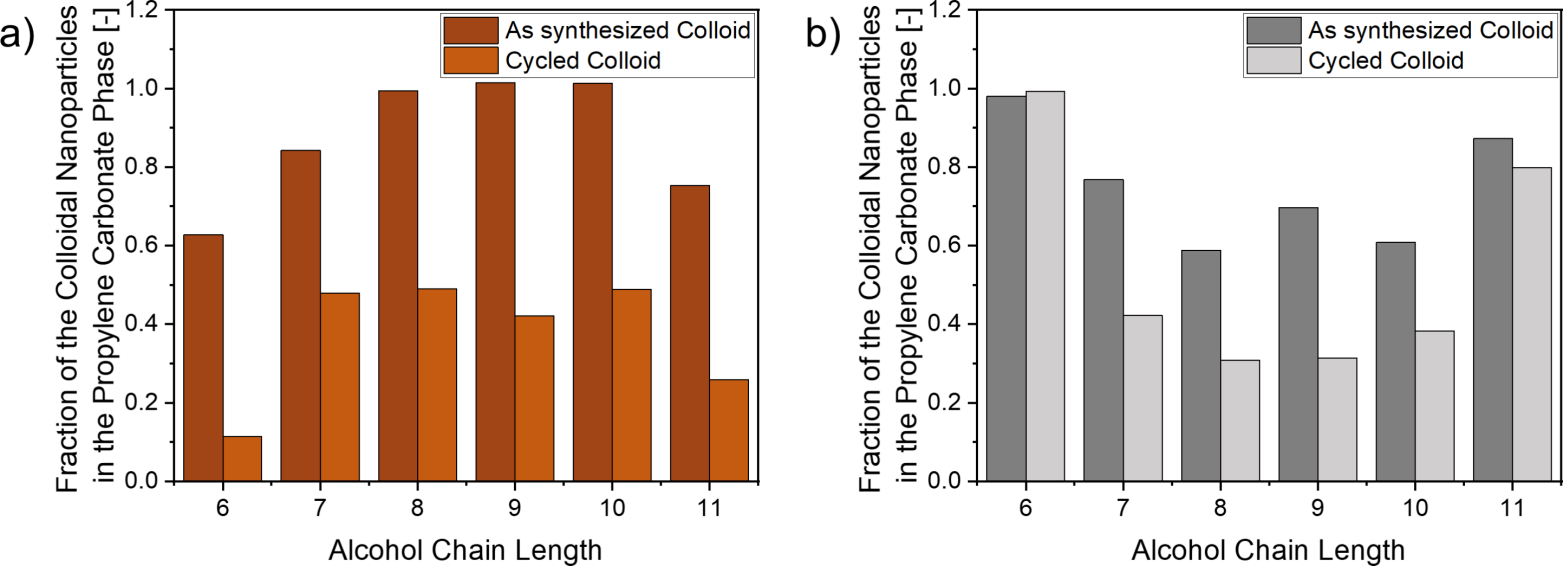
Mass fraction of colloidal nanoparticles in the propylene carbonate phase gained after LAL of (a) copper and (b) iron in TMSs consisting of propylene carbonate and a primary alcohol with a chain length of 6 to 11. The nanoparticle fraction is shown before and after cycling in the monophasic state.

In addition to the effect of the metal used in the LAL process on the preference of the laser-generated nanoparticles to PC or the alcohol phase, we further studied the effect of polarity of the used alcohol phase along a homologous series of alcohols with chain lengths ranging from C_6_ to C_11_. Given that Cu and Fe were the most sensitive for the PC/alcohol system we concentrated on these elements in the following study. To equilibrate the particle distribution, we cycled between the mono- and biphasic state three times. After cycling, the distribution of colloidal copper and iron nanoparticles over the PC and alcohol phase was quantified via UV–vis extinction measurements afterward (Figure 4). Figure 4 shows the mass fraction of colloidal nanoparticles in the propylene carbonate phase (bottom phase of the TMS) for the respective alcohols before and after cycling into the monophasic state. For the as-synthesized colloid, the fraction of copper nanoparticles in the bottom phase (PC) was close to 100% for C_8_ to C_10_, with a lower fraction of nanoparticles in the 1-hexanol, 1-heptanol, and 1-undecanol experiments. After cycling, the fraction drops to 11% for 1-hexanol and 26% for 1-undecanol. Interestingly, the fraction in the PC phase reaches a maximum between 1-heptanol and 1-decanol at around 50%. Finally, the fraction drops to 26% again for 1-undecanol. Apparently, the solvent properties of the C_7_ to C_10_ alcohols (with 1-nonanol being an exception) pose a sweet spot for the “solubility” of LAL-synthesized copper nanoparticles. In this regard, the solubility of the solvents in each other cannot be neglected. The solubility of alcohol in PC and vice versa is highest for 1-hexanol and decreases with increasing chain length. Hence, the amount of 1-undecanol is the lowest in PC and that of 1-hexanol is the highest in PC. The sweet spot might be linked to an optimal mixture of the two solvents in both phases, resulting in an enhanced enrichment of copper nanoparticles in the propylene carbonate phase. In contrast, iron nanoparticles were found to show the opposite trend with the sweet spot being a minimum for 1-octanol and 1-nonanol (instead of a maximum for Cu), which again confirms copper and iron as a turning point regarding their standard electrochemical reduction potential. The optimal mixing of the two solvents in both phases could be related to two possibilities. First, the mixing of the solvents affects the phase separation, which was previously associated with a decrease in colloidal stability if the phase separation was too fast. The mixing of the solvents can lead to a faster or slower phase separation which, in turn, can lead to different physical effects on the particles (such as, e.g., agglomeration). Second, it is possible that the nanoparticles inherently prefer a solvent polarity that lies between the polarity of the alcohol and propylene carbonate phases. Rather than a competitive phase separation process, the now mixed solvents would equilibrate to a medium polar solvent mixture preferred by the nanoparticles. However, as the Cu NP fractions are roughly the same for 1-heptanol, 1-octanol, and 1-decanol, this might not be restricted to polarity only (in case the Cu fraction in 1-nonanol is not an outlier). This effect seems to be pronounced more strongly if the standard electrochemical reduction potential is close to 0 V and results in an inversion in phase preference when the potential switches from, for example, a few +100 mV to a few −100 mV. However, this explanation is only a hypothesis and requires further investigation and computational simulations to verify. Furthermore, although this is beyond the scope of this study, it will be interesting to see how this trend changes when composition series of binary or multinary alloys with different average standard potential values close to 0 V are used during ablation in such TMS systems.

## Conclusion

**Figure 5:**
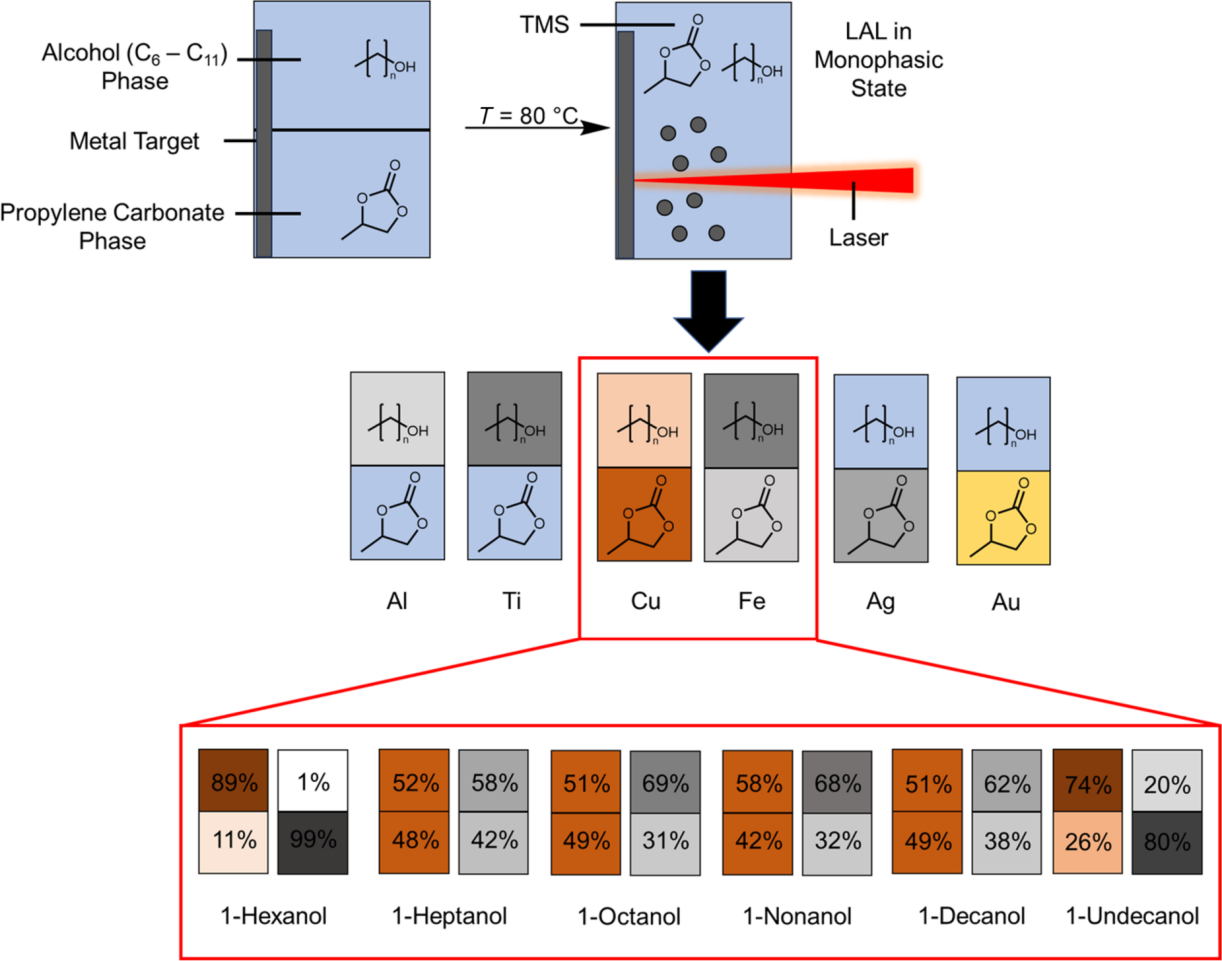
Schematic representation of the thermomorphic solvent systems investigated in this study. The upper part shows the synthesis pathway of the nanoparticles by LAL in an alcohol–propylene carbonate mixture, resulting in different nanoparticle phase preferences depending on the ablated metal. In the intermediate cases (iron and copper), the phase preference of the respective alcohol–propylene carbonate mixture with alcohol chain lengths of six to eleven carbon atoms is also given with the fractions given for each TMS. The intensity of the colors reflects the nanoparticle fraction in the respective phase.

While the research activities on LAL in solvents have highlighted the importance and role of chemical reactions on particle size and chemical functionalization (e.g., carbon shell) of the gained colloids, the influence of a mixture of two liquid solvents on the colloidal stability and selective enrichment of the nanoparticles in either of these phases has not yet been studied. Hence, in this study, six metals with different standard electrochemical reduction potential were ablated in a heated monophasic system consisting of propylene carbonate and an alcohol from the homologous series of primary alcohols (C_6_–C_11_). After cooling the gained colloids, the propylene carbonate and alcohol phases separated and the mass concentration of the colloidal nanoparticles in the respective phases was quantified. The gained colloids of gold and silver were found to preferentially reside in the more polar propylene carbonate phase, while aluminum and titanium nanoparticles were in the less polar alcohol phase. Copper and iron nanoparticles were found in both phases and appeared to represent the turning point. In the case of copper, whose standard electrochemical reduction potential is slightly positive, but close to zero, the copper nanoparticles were slightly more abundant in the propylene carbonate phase. The opposite was the case for iron whose standard electrochemical reduction potential is slightly negative. The tunability of behavior for copper and iron was further studied by varying the chain length of the used alcohols. It turned out that copper was particularly drawn to the PC phase when a C_7_–C_10_ alcohol was used. While copper nanoparticles remained abundant in PC in all cases, their concentration in the alcohol phase increased when the carbon chain length of the chosen alcohol phase was shorter (C_6_) or longer (C_11_). This behavior was mirrored when iron nanoparticles were synthesized in the same systems. Here, also more than 30% of the iron nanoparticles were present in PC in all cases but the abundance in PC was particularly low when the experiments were conducted with the C_8_ or C_9_ alcohol. Increasingly more iron enriched in the PC phase when alcohols with shorter (C_6_–C_7_) or longer (C_10_–C_11_) carbon chains were used. From a practical viewpoint, a few general points may be extracted (Figure 5). First, the phase preference of laser-generated nanoparticles in the TMS strongly depends on the standard electrochemical reduction potential of the metal. Higher standard electrochemical reduction potentials favor the more polar phase, while base metals accumulate in the less polar phase. Second, when reaching the standard electrochemical reduction potential close to zero, the former general behavior reaches a turning point, and the respective nanoparticle fractions (here of Fe and Cu) in each phase can be tuned by the carbon chain length of the alcohol phase. Among the investigated chain lengths of C_6_ to C_11_, 1-octanol and 1-nonanol provided the highest phase selectivity, showing either a maximum (for copper) or minimum (for iron) amount of metal nanoparticles in the phases. However, the system seems not to be in full equilibrium after the ablation process as repeated switching between the mono- and biphasic states shifted the mass concentration. For copper, the C_7_-C_10_ alcohols achieved the highest recovery rates in the PC phase, whereas, for iron, 1-hexanol or 1-undecanol would be the best choice to recover the nanoparticles in the PC phase during TMS cycling. Pinpointing the exact reason for this behavior requires a detailed investigation of the nanoparticles’ surface chemistry, composition, and crystal structure, ideally complemented by cycling studies after catalysis experiments. Overall, this study, however, provides first evidence that the phase selectivity and recyclability of nanoparticles fabricated in TMS are dictated by the standard electrochemical reduction potential of the material and the chain length of the semipolar component.

## Experimental

### Materials

Gold, silver, copper, iron, aluminum, and titanium substrates were used as ablation targets. Experiments were performed in propylene carbonate (99%, Carl Roth), glycerol carbonate (98.5%, Huntsman Products GmbH), 1-hexanol (99%, Fisher Scientific), 1-heptanol (98%, Sigma-Aldrich), 1-octanol (99%, Acros Organics), 1-nonanol (99%, TCI Deutschland GmbH), 1-decanol (99%, Sigma-Aldrich), 1-undecanol (98%, TCI Deutschland GmbH).

### Methods

Nanoparticles are generated by ablating the target material (10 × 10 × 1 mm) immersed in the organic solvent using a Nd:YAG-laser (RSM 100D, ROFIN-SINAR Laser GmbH) working with a pulse duration of 35 ns, a wavelength of 1064 nm, a repetition rate of 5 kHz and pulse energy of 5.4 mJ. LAL was performed in a 30 mL batch vessel filled with a volumetric ratio of 50% of propylene carbonate and the respective alcohol. The experiments conducted for Figure 4 were performed in a batch vessel that was heated with an optimized coating to enable uniform heating of the TMS and is depicted in Supporting Information File 1, Figure S3. The temperature of the batch vessel was set to 85 °C to induce a conversion to the monophasic TMS. Afterward, the target material was ablated for 10 min followed by storage of the batch vessel at room temperature for two hours to attain phase separation. A slow cooling rate was chosen to avoid precipitation of the nanoparticles at the phase boundaries.

Heating cycles were performed in a 50 mL vessel, which was heated up to 85 °C with a temperature ramp of 5 °C/min and temperature was held for 15 min. For the cooling of the colloid to room temperature, the heating was turned off and the vessel was held in place for two hours.

Colloids were characterized by UV–vis-spectroscopy using a Cary 50 spectrometer (Varian Inc.) and further processed by OriginPro. The raw UV–vis extinction spectra were baseline-corrected by subtraction of UV–vis extinction spectra gained from nanoparticle-free TMS (Supporting Information File 1, Figure S4c) from the UV–vis extinction spectra of the gained colloids in the respective TMS (Supporting Information File 1, Figure S4a,b). Fractions of the colloids in the respective phases were calculated by dividing the extinctions obtained from UV–vis-spectroscopy (Supporting Information File 1, Figure S1 and Figure S5) at the wavelength of the plasmon resonance peak (for Au and Ag) or at the wavelength of 550 nm (for Cu, Fe, Al, and Ti). The extinction at a wavelength of 550 nm for copper was used because the plasmon resonance peaks were not always detectible. Extinction values for the Cu and Fe colloids can be found in Supporting Information File 1, Table S1 and Table S2, and the fractions of colloidal NPs in the propylene carbonate phase are found in Supporting Information File 1, Table S3. High-resolution transmission electron microscopy (HRTEM) pictures were taken at a JEOL 2200FS (JEOL Ltd.) and further processed by ImageJ.

## Supporting Information

Additional material and data to support the results of the article. The data includes UV–vis extinction spectra of all gained colloids, zeta potential measurements, and a photograph of the used batch vessel.

File 1 Additional figures and tables.

## Data Availability

Data generated and analyzed during this study is available from the corresponding author upon reasonable request.
