# Supplementary material for: Preferential enrichment and extraction of laser-synthesized nanoparticles in organic phases
Source: Beilstein J Nanotechnol. 2025 Feb 20;16:254–63. doi: 10.3762/bjnano.16.20 (PMC11849555; doi:10.3762/bjnano.16.20)
Supplement: File 1 — Additional figures and tables. [file Beilstein_J_Nanotechnol-16-254-s001.pdf]

BEILSTEIN JOURNAL OF NANOTECHNOLOGY

Supporting Information

for

Preferential enrichment and extraction of laser-synthesized nanoparticles in organic phases

Theo Fromme, Maximilian L. Spiekermann, Florian Lehmann, Stephan Barcikowski, Thomas Seidensticker and Sven Reichenberger

Beilstein J. Nanotechnol. **2025,** *16,* 254–263. doi:10.3762/bjnano.16.20

Additional figures and tables

License and Terms: This is a supporting information file under the terms of the Creative Commons Attribution License (https://creativecommons.org/licenses/by/4.0). Please note that the reuse, redistribution and reproduction in particular requires that the author(s) and source are credited and that individual graphics may be subject to special legal provisions.
The license is subject to the *Beilstein Journal of Nanotechnology* terms and conditions: (https://www.beilstein-journals.org/bjnano/terms)

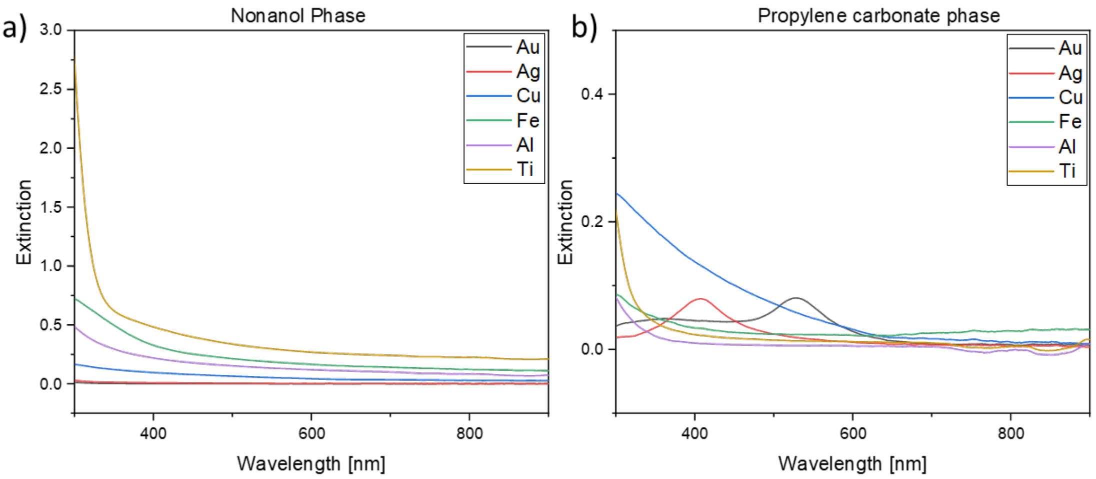

Figure S1: UV–vis extinction spectra of the gained colloids from the six different metals (Au, Ag, Cu, Fe, Al, and Ti) in the TMS consisting of propylene carbonate and 1-nonanol.

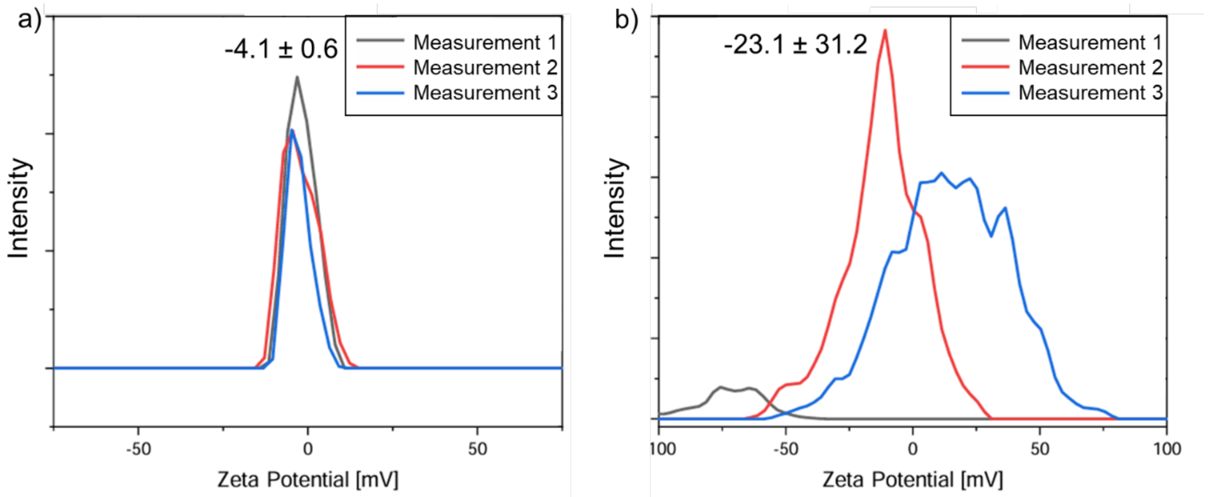

Figure S2: Zeta potential of iron nanoparticles in (a) 1-nonanol and (b) propylene carbonate obtained by LAL in the TMS of 1-nonanol and propylene carbonate.

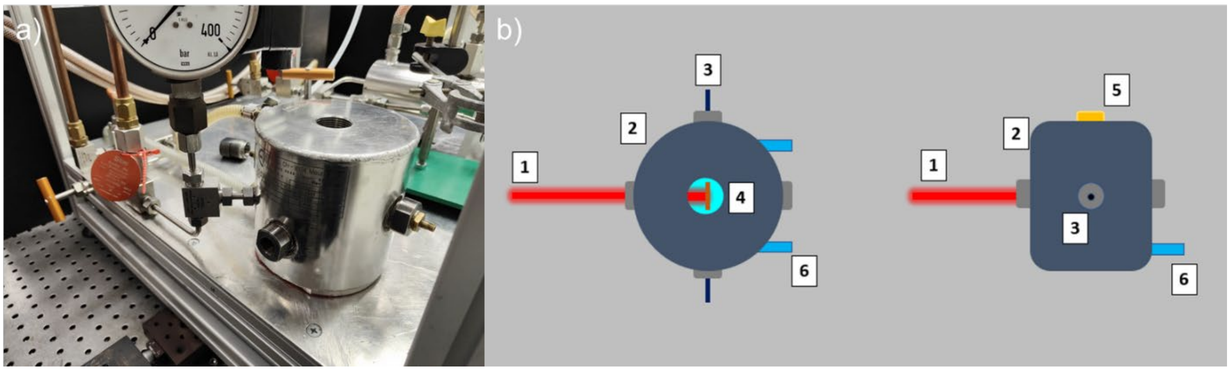

Figure S3: (a) Photograph of the batch vessel with a heating coat. (b) Schematic depiction of the batch vessel. The numbers indicate the following: 1 laser beam, 2 ablation chamber with a heating coat, 3 fiber UV–vis connector (not used in this study), 5 cover, 6 heating water connectors.

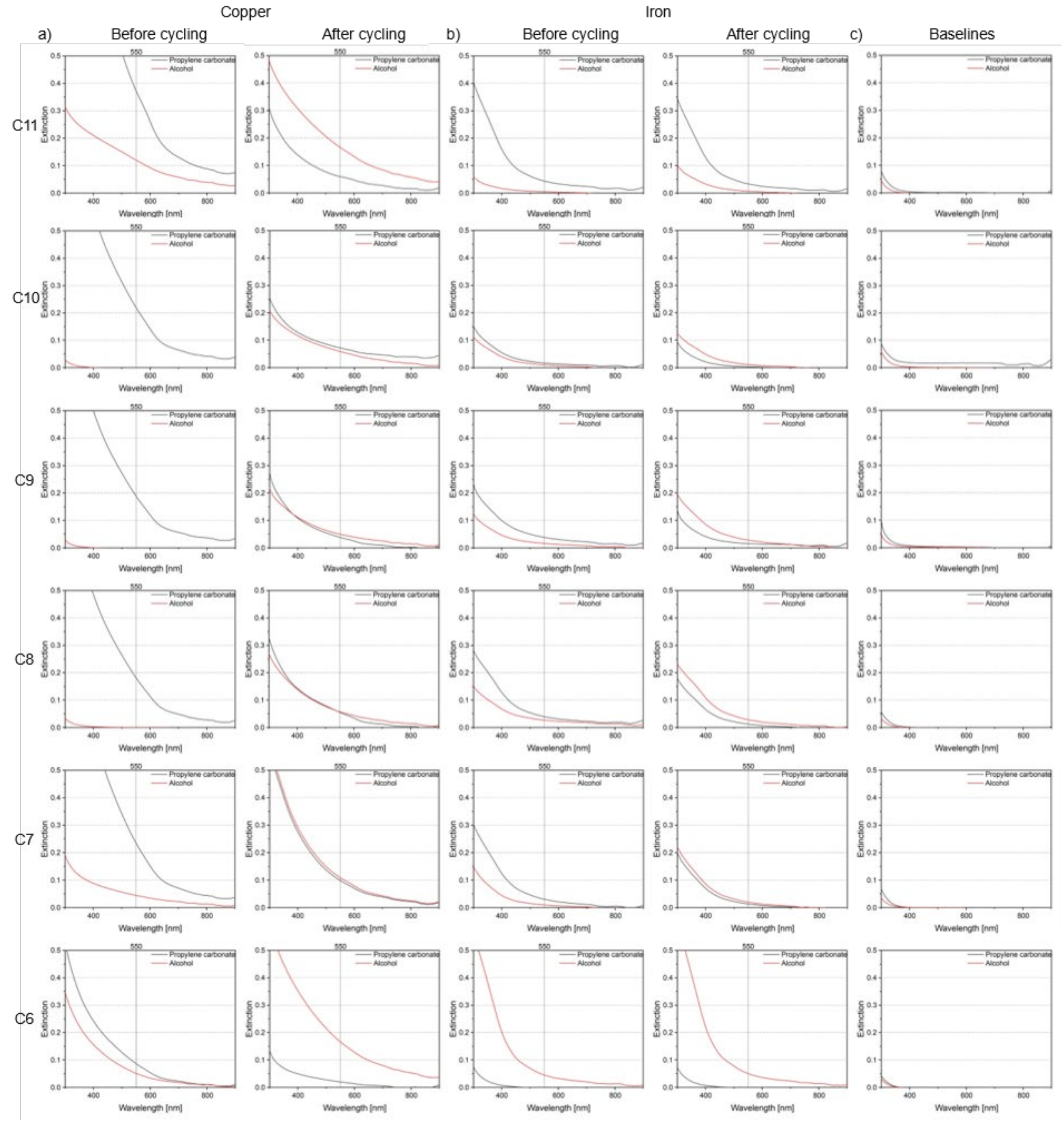

Figure S4: Raw UV–vis extinction spectra gained for (a) copper and (b) iron colloids in TMSs consisting of propylene carbonate and an alcohol with a carbon chain length between six and eleven before and after cycling. (c) UV–vis extinction spectra baselines gained from TMSs consisting of propylene carbonate and an alcohol with a carbon chain length of six to eleven after phase separation from the monophasic state.

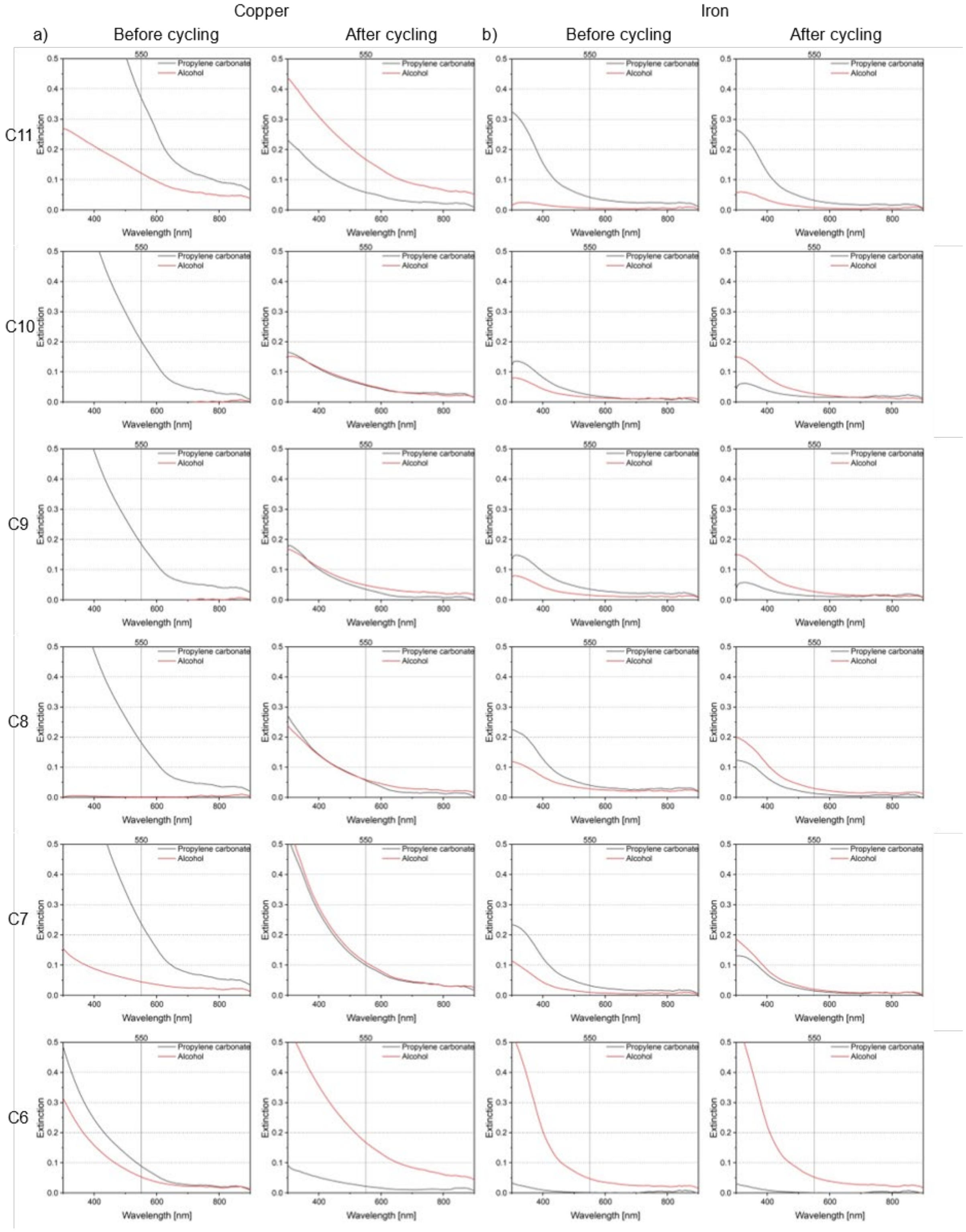

Figure S5: Baseline-corrected UV–vis extinction spectra gained for (a) copper and (b) iron colloids in TMSs consisting of propylene carbonate and an alcohol with a carbon chain length of six to eleven before and after cycling. The baseline-corrected UV–vis extinction spectra were obtained by subtraction of the respective baselines (Figure S4c) from the UV–vis extinction spectra of the raw colloids (Figure S4a,b).

Table S1: Extinctions at a wavelength of 550 nm gained from the baseline-corrected UV–vis extinction spectra (Figure S5) of the copper colloids in TMSs consisting of propylene carbonate and an alcohol with a carbon chain length of six to eleven before and after cycling.

Alcohol chain length	Extinction at 550 nm of the propylene carbonate phase [-]		Extinction at 550 nm of the alcohol phase [-]	
	before cycling	after cycling	before cycling	after cycling
11	0.371	0.058	0.121	0.167
10	0.203	0.055	0	0.057
9	0.185	0.035	0	0.048
8	0.182	0.056	0.001	0.058
7	0.238	0.100	0.044	0.109
6	0.089	0.021	0.052	0.168

Table S2: Extinctions at a wavelength of 550 nm gained from the baseline-corrected UV–vis extinction spectra (Figure S5) of the gained iron colloids in TMSs consisting of propylene carbonate and an alcohol with a carbon chain length of six to eleven before and after cycling.

Alcohol chain length	Extinction at 550 nm of the propylene carbonate phase [-]		Extinction at 550 nm of the alcohol phase [-]	
	Before cycling	After cycling	Before cycling	After cycling
11	0.042	0.031	0.006	0.008
10	0.023	0.016	0.015	0.027
9	0.035	0.012	0.015	0.027
8	0.040	0.013	0.028	0.029
7	0.032	0.014	0.009	0.019
6	0.046	0.052	9.7E-4	3.69E-4

Table S3: Fractions of the colloidal Cu and Fe NPs in the propylene carbonate phase of TMSs consisting of propylene carbonate and an alcohol with a chain length of six to eleven before and after cycling.

Alcohol chain length	Fraction of colloidal Cu NP in the propylene carbonate phase [-]		Fraction of the colloidal Fe NP in the propylene carbonate phase [-]	
	before cycling	after cycling	before cycling	after cycling
11	0.75	0.26	0.87	0.80
10	1.00	0.49	0.60	0.38
9	1.00	0.42	0.69	0.32
8	0.99	0.49	0.59	0.31
7	0.84	0.48	0.77	0.42
6	0.62	0.11	0.98	0.99
